# Surgical Diseases of the Ovaries and Fallopian Tubes, Including Tubal Pregnancy

**Published:** 1893-03

**Authors:** 


### Reviews of Books.

*Surgical Diseases of the Ovaries and Fallopian Tubes, including Tubal Pregnancy.* By J. BLAND SUTTON, F.R.C.S. Pp. xvi., 500. London: CASSELL & COMPANY, LIMITED. 1891.

This book is not one of those which are simply collections of statements made by various writers. It is full of original observation. It is the book of a man who has thoroughly studied his subject, who is constantly seeking for new facts from which to form deductions, and seeking in all directions, from comparative as well as human anatomy and pathology, and who has a wide field of observation, not only in the sphere of his own work, but from numerous specimens sent to him by others. The book is the outcome of much laborious and very careful investigation. It is very clearly written, and everywhere just to the point. It is profusely illustrated, and the pictures are exceedingly good. The coloured plates are excellent, and all the drawings very clear.

The clinical portion of the work rests on a very complete basis of pathology. Again and again we are told at the present day that it is sufficient for surgeons to know how to diagnose their patients' ailments, and how best to treat them. This is called the practical aspect of surgery. Pathology is spoken of as the scientific side of the work, concerning which we may acquire some information if we have time. But what is pathology but a knowledge of the condition of things we have to diagnose and treat as practical surgeons? It is not some collateral science, as we might suppose from its frequent dissociation from diagnosis and treatment. It is by careful and thorough work in the *post-mortem* room, and examination of morbid growths removed during life, that we shall gain a knowledge of disease of immense practical value when we have to diagnose and to operate; and of no department of work is this more true than of ovarian disease. This is why a work of this kind—a work with a sound pathological basis—is so valuable as a practical guide.

The book is divided into four parts. The first treats of diseases of the ovary; the second, diseases of the Fallopian tubes; the third, tubal pregnancy; and the fourth, the methods of performing operations for ovarian and tubal disease.

Mr. Sutton believes, as the result of numerous observations, that the mucous membrane is not shed at menstrual times, as is usually taught. As might have been expected from the author's previous book on the subject, there is a particularly good section on dermoid cysts of the ovary, the drawings of which are a very interesting series. Mr. Sutton holds strongly the view that dermoids found growing on the omentum or mesentery have been originally ovarian; that they contract adhesions to the mesentery, and then their attachment to the ovary gets drawn out and thinned, and eventually gives way. And he tells us that he has seen these cysts attached in this way to both. Dermoid cysts of the ovary are usually regarded as quite innocent growths. Bland Sutton believes that the majority are; but he quotes Thornton to the effect that in some cases, where sarcoma tissue was found in ovarian dermoids, solid sarcomata recurred in the pelvis after their removal. In another case of Jessop's, the cyst contained carcinomatous growth, and there were secondary growths in the liver. We must not, he tells us, however, too readily conclude that there is sarcoma tissue in these cysts, as so many tissues are represented in them. He mentions the great length to which the hair grows in some of these dermoids, and alludes to a specimen now in the Museum of the Bristol Children's Hospital, in which is a lock of hair 28 inches in length.

It is, of course, well known that cysts arising in the hilum of the ovary, where remains of the Wolffian body are to be found, very often contain papillomatous growth, and that ordinary multilocular cysts arising in the oöphoron of the ovary do not. Bland Sutton, however, describes small cysts in the oöphoron containing warty growths; but these, he says, never attain any size, and the warty growths in them are of almost cartilagious hardness.

We do not think that Mr. Sutton has made out a case for grouping ovarian tumours in children as a class by themselves, but the limitation of space prevents our going into this question in detail.

The chapters on extra-uterine gestation are of particular value and interest. Mr. Sutton has devoted much time and attention to this subject, and has thrown much light on the real nature of many cases described simply as tubal or broad-ligament hæmatocele. There is no subject about which it is more important to have clear ideas with regard to diagnosis and treatment, for at any time we may be suddenly called upon to try and save the life of a woman who is in great danger from a ruptured extra-uterine gestation; and the information given by Bland Sutton will be found invaluable.

The book, as a whole, is one that will be read with the greatest interest and the greatest profit, both by the gynæcologist and the abdominal surgeon.

